# Glial expression of Swiss cheese (SWS), the *Drosophila* orthologue of neuropathy target esterase (NTE), is required for neuronal ensheathment and function

**DOI:** 10.1242/dmm.022236

**Published:** 2016-03-01

**Authors:** Sudeshna Dutta, Franziska Rieche, Nina Eckl, Carsten Duch, Doris Kretzschmar

**Affiliations:** 1 Oregon Institute of Occupational Health Sciences, Oregon Health & Sciences University, Portland, OR 97239, USA; 2 Institut für Zoologie III – Neurobiologie, Universität Mainz, Colonel-Kleinmann-Weg 2, Mainz D-55099, Germany

**Keywords:** Ensheathing glia, Axonal degeneration, Phospholipase, PNPLA6, Spastic paraplegia

## Abstract

Mutations in *Drosophila* Swiss cheese (SWS) or its vertebrate orthologue neuropathy target esterase (NTE), respectively, cause progressive neuronal degeneration in *Drosophila* and mice and a complex syndrome in humans that includes mental retardation, spastic paraplegia and blindness. SWS and NTE are widely expressed in neurons but can also be found in glia; however, their function in glia has, until now, remained unknown. We have used a knockdown approach to specifically address SWS function in glia and to probe for resulting neuronal dysfunctions. This revealed that loss of SWS in pseudocartridge glia causes the formation of multi-layered glial whorls in the lamina cortex, the first optic neuropil. This phenotype was rescued by the expression of SWS or NTE, suggesting that the glial function is conserved in the vertebrate protein. SWS was also found to be required for the glial wrapping of neurons by ensheathing glia, and its loss in glia caused axonal damage. We also detected severe locomotion deficits in glial *sws*-knockdown flies, which occurred as early as 2 days after eclosion and increased further with age. Utilizing the giant fibre system to test for underlying functional neuronal defects showed that the response latency to a stimulus was unchanged in knockdown flies compared to controls, but the reliability with which the neurons responded to increasing frequencies was reduced. This shows that the loss of SWS in glia impairs neuronal function, strongly suggesting that the loss of glial SWS plays an important role in the phenotypes observed in the *sws* mutant. It is therefore likely that changes in glia also contribute to the pathology observed in humans that carry mutations in *NTE*.

## Abstract (precis)

***Drosophila* Collection:** Loss of *sws* in glia results in locomotion deficits, suggesting that glial changes contribute to the paralysis and spastic paraplegia in humans carrying mutations in its orthologue, *NTE*.

## INTRODUCTION

*swiss cheese* (*sws*) mutant flies show degeneration of the adult nervous system that is detectable around day 5 of adulthood by the formation of spongiform lesions in the brain (Kretzschmar et al., 1997). This phenotype progresses with age and the flies die prematurely. SWS is an evolutionarily conserved protein, which, in mammals, is called neuropathy target esterase (NTE) or patatin-like phospholipase domain containing 6 (PNPLA6) (Lush et al*.*, 1998). NTE was first linked to neuronal degeneration in the 1930s, when thousands of people were paralyzed after consuming a beverage (Jamaica Ginger) that contained the organophosphorus compound tri-ortho-cresyl phosphate (TOCP). TOCP and other organophosphates bind to and interfere with NTE's lipase activity, leading to a delayed neuropathy called organophosphate-induced delayed neuropathy (OPIDN) (Johnson, 1969). However, NTE is not only involved in this environmentally induced neuronal degeneration but also causes an inherited spastic paraplegia (spastic paraplegia 39) when mutated, a condition that is characterized by progressive weakness of the upper and lower limbs (Rainier et al., 2008). In addition, mutations in NTE have recently been identified in individuals with Boucher-Neuhäuser and Gordon-Holmes syndromes, complex disorders that can include hypogonadism, chorioretinal dystrophy, cerebellar atrophy and cognitive impairment (Deik et al., 2014; Synofzik et al., 2014a,b; Topaloglu et al., 2014). Furthermore, NTE mutations can cause retinal degeneration during childhood (e.g. Oliver McFarlane syndrome) (Kmoch et al., 2015).

*NTE*-knockout mice show impaired vasculogenesis and placental defects, leading to lethality around day 9 postcoitum (Moser et al., 2004). In contrast, a brain-specific knockout is viable but the animals show neuronal death and defects in motor coordination when aged (Akassoglou et al., 2004). *NTE* expression is detectable in the nervous system and the spinal ganglia starting around day 13 postcoitum (Moser et al., 2000). Postnatally, *NTE* is widely expressed in the brain but becomes more restricted during aging with a strong expression in large neurons in the cortex, olfactory bulb, thalamus, hypothalamus, pons, and medulla oblongata (Glynn et al., 1998; Moser et al., 2000). Similarly, we previously found *sws* to be widely expressed in the brain, with most or all neurons containing SWS (Muhlig-Versen et al., 2005). SWS shares a highly conserved esterase domain with NTE that mediates the phospholipase activity and contains the binding site to which organophosphates bind (Glynn, 2013; Muhlig-Versen et al., 2005; Quistad et al., 2003). Like in vertebrates, organophosphate treatment induces degeneration and locomotion deficits in flies (Wentzell et al., 2014). In addition, both NTE and SWS have several cyclic-nucleotide-binding sites (Lush et al., 1998; Moser et al., 2000), and a domain that can bind to and inhibit the PKA-C3 catalytic subunit of Protein kinase A (Bettencourt da Cruz et al., 2008). Both SWS and NTE can bind to and inhibit the activity of PKA-C3, and this domain is necessary to prevent neuronal degeneration in flies (Bettencourt da Cruz et al., 2008; Wentzell et al., 2014). However, SWS is also expressed in glia (Muhlig-Versen et al., 2005), and *sws* mutant flies show glial hyperwrapping and glial death (Kretzschmar et al., 1997). The idea that SWS is autonomously required in glia was suggested by experiments expressing SWS specifically in neurons in *sws* mutants, which suppressed the neuronal degeneration but not the glial phenotypes (Muhlig-Versen et al., 2005). *Vice versa*, expressing SWS in glia prevented the glial defects but not the neuronal degeneration. Expressing mouse NTE in glia or neurons in *sws* mutants had the same effect (Muhlig-Versen et al., 2005), strongly suggesting that both the neuronal and the glial functions are important conserved features of these proteins. However, neither the specific glial subtype that requires SWS nor the effects on neuronal function and how this contributes to the deficits observed in *sws* mutant flies, and possibly in patients, were known.

## RESULTS

### Loss of glial SWS leads to abnormal glial morphology and death

Our previous characterization of the *sws^1^* mutant, in which no SWS protein could be detected, showed that the loss of SWS resulted in the formation of membranous glial structures, especially in the lamina cortex, and glial cell death (Kretzschmar et al., 1997). Like the neuronal degeneration, this phenotype was progressive, with these structures becoming larger and more numerous with age. These structures were very prominent in the lamina but we occasionally also found some in other brain areas and, in young flies, we observed multiple glial sheaths around axons and neuronal cell bodies (Fig. S1B,C,F,G). Intriguingly, however, other neurons revealed an incomplete glial wrapping (Fig. S1B,D). To address whether these phenotypes are caused by the loss of SWS in glia, we used a knockdown approach. To achieve a knockdown of SWS in all glia, we induced the *sws^GD3277^* RNAi line with the pan-glial *loco*-*GAL4* or *repo*-*GAL4* driver lines (Granderath et al., 1999; Jefferis et al., 2004). Both drivers resulted in similarly reduced overall SWS protein levels (Fig. S2). Owing to SWS still being expressed in neurons in these flies, the observed reduction in the overall levels of SWS suggests a substantial knockdown of SWS in glia.

**Fig. 1.**
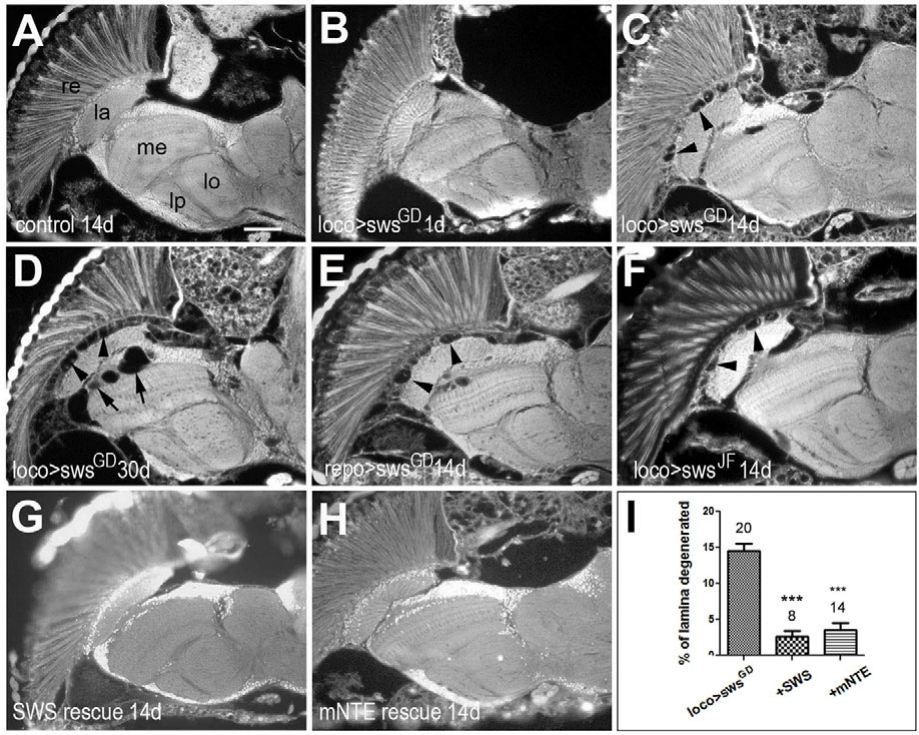
**The glial knockdown of** **SWS causes progressive vacuolization.** (A) Paraffin head section from a 14-day-old (14 d) *loco*-*GAL4;UAS-lacZ* control fly does not reveal histological defects. Knocking down SWS using *loco*-*GAL4* and *UAS-sws^GD3277^* (sws^GD^) in all glia does not cause detectable phenotypes when 1 day old (B) but leads to increasing vacuolization, especially in the lamina cortex (arrowheads), when 14 days (C) or 30 days (D) old. In the 30-day-old knockdown flies, we also detected vacuoles near the first optic chiasm (arrows). A similar phenotype as in the 14-day-old *loco-GAL4;UAS-sws^GD3277^* flies was detectable when driving *UAS-sws^GD3277^* with *repo*-*GAL4* (E) or when using *loco*-*GAL4* and *UAS-sws^JF03428^* (sws^JF^; F) of 14-day-old flies. (G,I) Co-expressing *Drosophila* UAS-SWS with sws^GD^ using *loco*-*GAL4* almost completely prevented the vacuolization in 14-day-old flies. (H,I) Similarly, murine NTE (mNTE) rescues the phenotype when co-expressed with sws^GD^ in glia cells (via *loco*-*GAL4*; 14 days old). (I) Quantification of the lamina degeneration in 14-day-old knockdown and rescue flies. The number of analyzed laminae and the s.e.m. are indicated for each bar. ***P<0.001. re, retina; la, lamina; me, medulla; lo, lobula; lp, lobula plate. Scale bar: 25 µm.

To determine whether the knockdown of SWS in glia causes the membranous structures in the lamina cortex, we first analyzed paraffin-embedded head sections. We previously observed that the membranous glial structures observed in plastic-embedded sections appear as vacuoles in paraffin sections, probably owing to being composed mainly of lipids, which are not well fixed in the paraffin sections. Analyzing 1-day-old *loco*-*GAL4;sws^GD3277^* flies (Fig. 1B), we did not detect any abnormalities compared to control flies expressing *lacZ* (as a control UAS construct) with this driver (not shown). However, at 14 days old, vacuoles had formed in the lamina cortex (arrowheads, Fig. 1C), a phenotype that was not detectable in age-matched *lacZ*-expressing control flies (Fig. 1A). At 30 days of age, the entire lamina cortex was filled with vacuoles (arrowheads, Fig. 1D) and additional lesions had formed near the first optic chiasm (arrows, Fig. 1D) at a position where the chiasm glial cells are localized (Tix et al., 1997). As expected, using the *repo*-*GAL4* driver to induce *sws^GD3277^* caused a similar vacuolization as was seen with *loco*-*GAL4* (Fig. 1E, 14 days old). To confirm that this phenotype is due to a knockdown of SWS and not some off-target effects of the *sws^GD3277^* RNAi construct [although no off targets are predicted for this construct; Vienna *Drosophila* Research Center (VDRC)], we used another RNAi line (*sws^JF03428^*) which again showed the formation of lesions in the lamina cortex when induced with *loco*-*GAL4* (Fig. 1F, 14 days). Finally, the vacuolization could be rescued by the co-expression of *sws* (Fig. 1G) or mouse *NTE* with *sws^GD3277^* (Fig. 1H), and the quantification of the vacuolization showed that there is no significant difference in the efficiency of the fly versus the mouse protein (Fig. 1I). These rescue experiments show that the phenotype in the knockdown flies is indeed due to the loss of SWS and that the vertebrate protein is capable of preserving glial function.

**Fig. 2.**
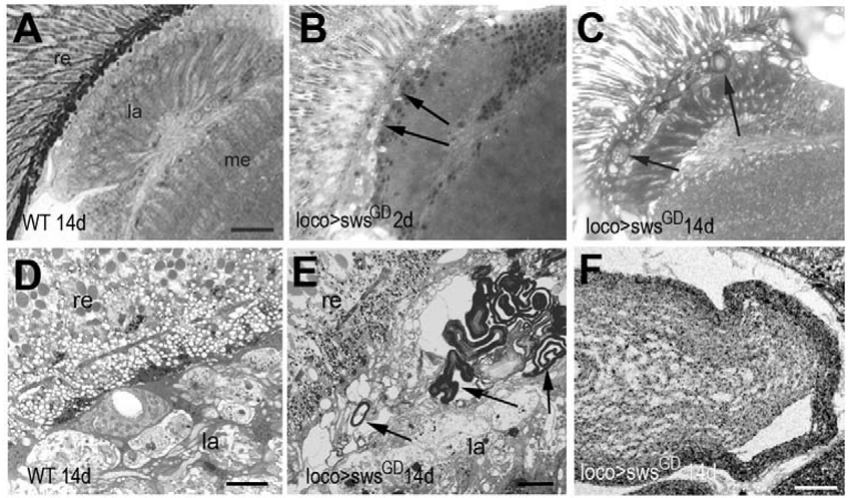
**The glial knockdown of SWS induces the formation of membranous glial bodies.** (A) A 1-µm plastic section reveals an intact lamina cortex in a 14-day-old (14 d) wild-type (WT) fly. (B) In 2-day-old *loco*-*GAL4;sws^GD^* animals, small membranous structures are detectable in the distal lamina cortex (arrows); these structures become larger when these flies are 14 days old (C, arrows). (D) Electron microscopic image showing the intact lamina cortex in a 14-day-old wild type. (E) A 14-day-old *loco*-*GAL4;UAS-sws^GD3277^* fly shows a highly disrupted lamina cortex and the formation of electron-dense multilayered membranous structures (arrows). A magnification of one of these structures is shown in F. Scale bar in A: 15 µm, in D,E: 2 µm, in F: 100 nm. re, retina; la, lamina; me, medulla.

To confirm that the vacuoles seen in the paraffin-embedded sections were due to the formation of the membranous structures, we performed analysis on semi-thin plastic-embedded sections. Indeed, we found that some of these membranous structures, although small, can already be detected in 2-day-old *loco*-*GAL4;sws^GD3277^* knockdown flies (arrows, Fig. 2B), and they become larger and more prominent in aged flies (Fig. 2C, 14 days old). The same glial whorls were detected in 14-day-old knockdown flies when using *repo*-*GAL4* (data not shown), whereas we did not detect them in age-matched controls (Fig. 2A). Finally, we performed electron microscopy on sections of 14-day-old *loco*-*GAL4;sws^GD3277^* knockdown flies and again detected the formation of membranous structures in the lamina cortex (Fig. 2E,F) that were not present in age-matched wild-type flies (Fig. 2D). This verifies that the loss of SWS in glia is responsible for the formation of the multi-layered membranous glial structures characteristic of the *sws* mutant.

To address the effects of the knockdown on glial survival, we performed immunohistochemical stainings using an anti-REPO antibody. Using this antibody, we could easily detect glial cells in 2-day-old (not shown) and 14-day-old control flies carrying only the *repo* driver (Fig. S3A). However, already in 2-day-old *repo*-*GAL4;sws^GD3277^* knockdown flies, their number was reduced (Fig. S3B, the arrowheads point to the epithelial glia) and this was even more prominent in 14-day-old knockdown flies (Fig. S3C). To quantify the loss of glial cells, we counted the immune-positive cells surrounding the optic lobes (excluding the lamina, because the vacuoles and resulting loose tissue complicated counting) or the epithelial glia cells in the lamina neuropil (arrowheads in Fig. S3B,C). Comparing 2-day- and 14-day-old control flies revealed no difference, showing that, within this time frame, aging was not affecting glial cell number (Fig. S3D,E). However, 2-day-old *repo*-*GAL4;sws^GD3277^* knockdown flies showed a clear reduction in glial cell numbers, with a reduction to 58% of the number of glial cells surrounding the optic lobes as compared with controls, and 75% of epithelial glial cells present when compared to controls (Fig. S3D,E). In 14-day-old knockdown flies, the number of the optic-lobe glia was further decreased to only 33%, which is not only significantly different from the age-matched control but also from 2-day-old knockdown flies (*P*<0.001). Counting the epithelial cells in 14-day-old knockdown flies still revealed a significant difference to age-matched controls but there was no difference between 2-day- and 14-day-old knockdown flies (Fig. S3D,E). To determine whether the glial death is due to apoptosis, we used an antibody against the apoptotic marker activated Caspase 3 in combination with anti-REPO. As shown in Fig. S3F (arrow), we could occasionally detect glial nuclei, in this case a giant glia cell of the outer chiasm (Tix et al., 1997), that were also positive for anti-activated Caspase 3 in 3- and 7-day-old knockdown flies, suggesting that at least some of the glial cells undergo apoptosis. Together, these experiments show that the loss of SWS leads to glial cell death, whereby the epithelial glia seem less dependent on SWS for survival than other glial cell types.

### SWS is required in subperineurial glia

**Fig. 3.**
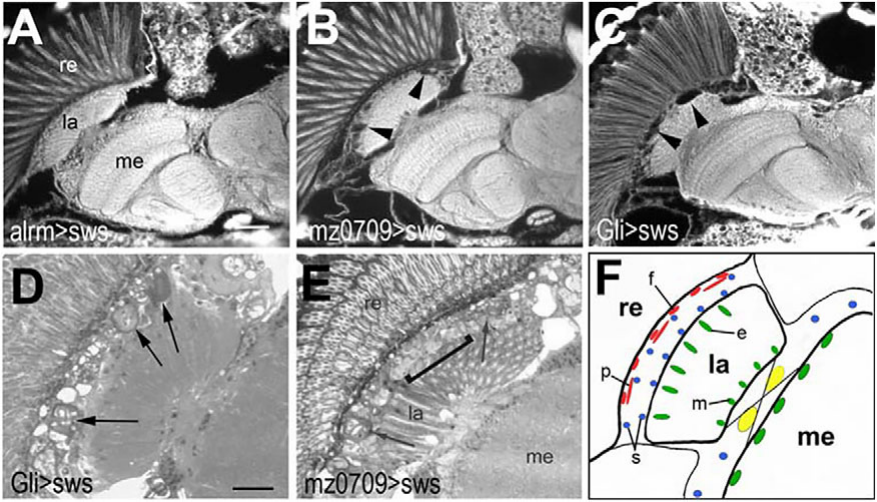
**SWS is required in lamina glia.** Paraffin head sections of 14-day-old flies show that inducing the *sws^GD^* RNAi construct with *alrm*-*GAL4* does not cause vacuolization in the lamina cortex (A), whereas the knockdown via *mz0709-GAL4* (B, arrowheads) or *Gli*-*GAL4* (C, arrowheads) does. Analyzing plastic sections, we found the membranous structures in 14-day-old *Gli*-*GAL4;sws^GD^* (D, arrows) and *mz0709-GAL4;sws^GD^* (E, arrows) flies, but the phenotype was weaker in the latter, with some parts of the lamina cortex still unaffected at this age (E, bracket). (F) Schematic showing the glial subtypes of the lamina. The fenestrated (f) and pseudocartridge (p) glia belong to the surface glia (red), the inner and outer satellite glia (s) to the cortex glia (blue), the epithelial glia (e) and marginal glia (m) to the neuropil glia (green). The chiasma glia is shown in yellow. Scale bar in A: 25 µm, in D: 12 µm. re, retina; la, lamina; me, medulla.

The results above suggested that different glial subpopulations react somewhat differently to the loss of SWS, and our previous immunohistochemistry experiments suggested that some glial cells might not express SWS (Muhlig-Versen et al., 2005). We therefore used glia-subtype-specific *GAL4* lines to identify glial subpopulations that are affected by the loss of SWS. The main types of glia in *Drosophila* are surface glia (perineurial and subperineurial glia), which provide the blood-brain barrier, cortex glia, which are mostly associated with neuronal cell bodies, and two types of neuropil glia: astrocyte-like glial cells and ensheathing glia (Awasaki et al., 2008; Edwards and Meinertzhagen, 2010; Freeman and Doherty, 2006; Lavery et al., 2007). To induce *sws^GD3277^* in astrocyte-like glia, we used the *alrm*-*GAL4* driver line (Doherty et al., 2009) but could not detect any defects in brain sections of 14-day-old flies (Fig. 3A). Similarly, we did not detect a phenotype when knocking down SWS in cortex glia via *NP2222* (Awasaki et al., 2008; data not shown). In contrast, the knockdown in ensheathing glia via *mz0709-GAL4*, which is also expressed in subperineurial glia (Ito et al., 1995), resulted in the formation of the characteristic lesions in the lamina cortex of 14-day-old flies (Fig. 3B) that we found in the pan-glial knockdown. Lastly, we used *Gli*-*GAL4*, which is a driver line for subperineurial glia, a subtype of surface glia that forms a blood-brain-barrier-like sheath around the CNS and peripheral nerves (Auld et al., 1995). Again, we detected vacuoles in the lamina cortex of 14-day-old knockdown flies (Fig. 3C), and the phenotype was stronger than with *mz0709-GAL4*. This was confirmed when we performed analysis on plastic-embedded head sections because we could easily detect the membranous glial structures along the whole length of the lamina cortex in 14-day-old *Gli-GAL4;sws^GD3277^* flies (arrows, Fig. 3D). Although these were also detected in 14-day-old *mz0709-GAL4;sws^GD3277^* flies (arrows, Fig. 3E), they were less abundant and some areas of the lamina cortex still looked fairly normal (bracket, Fig. 3E). The lamina cortex houses several glial subpopulations, including the pseudocartridge glia (‘p’ in Fig. 3F), which belongs to the subperineurial glia (Edwards and Meinertzhagen, 2010). Because both the knockdowns with *mz0709-GAL4* and *Gli-GAL4*, which share expression in subperineurial glia, resulted in the formation of the membranous structures in the lamina cortex (whereas *alrm*-*GAL4* and *NP2222* did not), this phenotype seems to be due to a loss of SWS in pseudocartridge glial cells.

### Loss of SWS results in loss of neurite ensheathment by glia

**Fig. 4.**
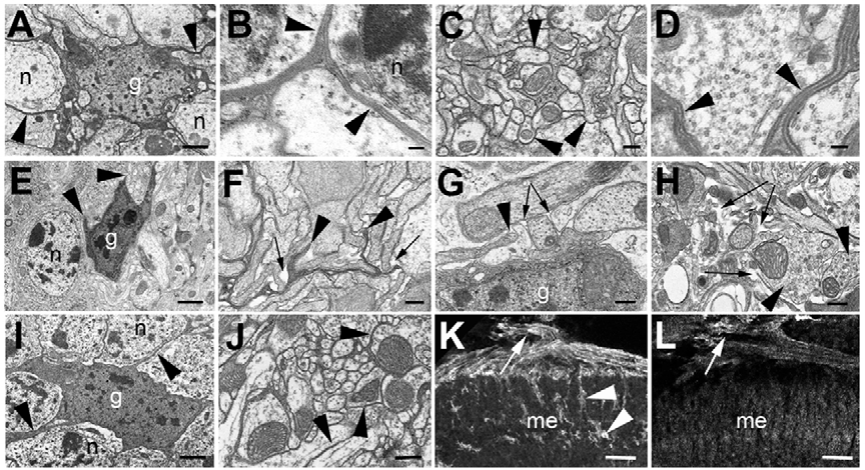
**Loss of glial wrapping in the glial knockdown.** (A-D) In a 14-day-old wild type, glial cells (g) extend processes (arrowheads) that enwrap neuronal cell bodies (n) (A,B) and neurites (C,D). (E,F) In a 14-day-old *mz0709-GAL4;sws^GD^* fly, glia show stunted processes (arrowheads) leading to a loss of glial ensheathment of neuronal cell bodies (n in E) and neurites (F). In addition, small empty spaces occur between neurites (arrows in F). (G) A glial cell body in a 1-day-old *repo*-*GAL4;sws^GD^* fly has only extended a short process (arrowhead), leading to incomplete wrapping of neurites and gaps between neurites (arrows). (H) When aged for 14 days, the spaces between neurites have widened (arrows) and many neurites are filled with vesicles (arrowheads). (I,J) Knocking down SWS with *alrm*-*GAL4* and *sws^GD^* did not result in detectable changes in glia or neurite morphology. Arrowheads point to glial processes. (K) Inducing mCD4-GFP with *mz0709-GAL4* reveals that this glial cell type sends processes into the neuropil (arrowheads; fly was 14 days old).  Arrow points to the first optic chiasm. (L) In a 14-day-old *mz0709 mCD4-GFP, GAL4;sws^GD^* fly these processes are not detectable and only processes along the first optic chiasm are still detectable (arrow). Scale bars in A,E,I: 1 µm, in B,D: 100 nm, in C,F-H,J: 0.5 µm, in K,L 10 µm. me, medulla.

*mz0709-GAL4* is not only expressed in subperineurial glia but also in the ensheathing neuropil glia throughout the brain, so we therefore investigated whether we can detect phenotypes of the glial knockdown in other brain areas. Because the lamina contains both glial types, we focused on the medulla and lobula for these experiments. In wild type, glial cells (which are more electron-dense) extend fine processes that form thin glial sheaths between cell bodies (arrowheads, Fig. 4A,B) resulting in the described complete glial wrapping of neuronal cell bodies (Buchanan and Benzer, 1993). Similarly, glial processes can be detected between neurites (arrowheads, Fig. 4C,D). In contrast, the glial processes in 14-day-old *mz0709-GAL4;sws^GD3277^* knockdown flies were often stunted (arrowheads, Fig. 4E,F), a phenotype that we did not detect in wild-type flies. Also, most neurites lacked the electron-dense glial sheaths and were less tightly packed than wild-type neurites, with gaps forming between them (arrows, Fig. 4F). The same phenotype occurred in 1-day-old *repo-GAL4;sws^GD3277^* flies, with stunted glial processes (arrowhead, Fig. 4G) and gaps showing between neurites (arrows). This was even more prominent in 14-day-old *repo-GAL4;sws^GD3277^* flies, where almost all neurites were surrounded by empty space (arrows, Fig. 4H). In addition, we observed various vesicular inclusions in these neurites (arrowheads, Fig. 4H). In contrast, 14-day-old *alrm-GAL4;sws^GD3277^* flies were indistinguishable from wild type and did not show this phenotype (Fig. 4I,J). At least in the antennal lobes, the ensheathing glia was originally described to extend processes around neuropils, but not into the neuropils (Doherty et al., 2009), and therefore the loss of ensheathment in the neuropil was somewhat surprising. We therefore expressed mCD4-GFP via *mz0709-GAL4* and used an antibody against GFP to determine whether this glial subtype extends processes into the optic neuropils. We confirmed that this glial cell type enwraps neuropils but we also found that it sends processes into the neuropils of the medulla (arrowheads, Fig. 4K), as well as into the lobula and lobula plate, and it ensheaths axons in the first and second optic chiasm (Fig. S4). As expected from results using the electron microscopic images, these GFP-positive processes were almost completely absent in 14-day-old *mz0709-GAL4;sws^GD3277^* flies and only in the first optic chiasm was some staining still detectable (arrow Fig. 4L).

Analyzing the *sws^1^* mutant also showed incomplete neuronal ensheathment but the more prominent phenotype was glial hyperwrapping. Intriguingly, the hyperwrapping was absent in the glial knockdown, suggesting that this is not a phenotype caused by the loss of SWS in glia. We therefore investigated whether this might be a non-autonomous effect on glia, caused by the loss of SWS in neurons, by knocking down SWS pan-neuronally using *Appl*-*GAL4* and *sws^GD3277^*. We did not detect any hyperwrapping in these flies when 1 day old (data not shown), an age at which we already found these structures in *sws^1^* mutants (Kretzschmar et al., 1997), or even when 14 days old (data not shown). That we did not observe glial hyperwrapping in either the neuronal knockdown or the glial knockdown might indicate that this phenotype only occurs when SWS is missing in both neurons and glia, as is the case in the *sws* mutant. This is also supported by rescue experiments inducing SWS in the *sws^1^* mutant, which showed that only the glial rescue restored the incomplete glial wrapping, whereas induction in either neurons or glia both rescued the hyperwrapping phenotype (Fig. S5).

### The phospholipase function of SWS plays a crucial role in glia

**Fig. 5.**
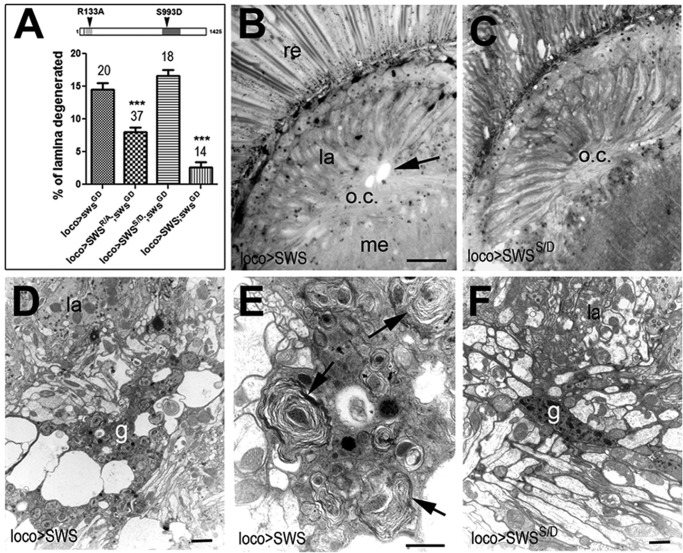
**The phospholipase domain is required for glial function.** (A) Expression of the SWS construct mutated in the PKA-C3 binding site (SWS^R/A^) and wild-type SWS significantly reduce the vacuolization in the lamina cortex of 14-day-old *loco*-*GAL4;sws^GD^* flies. In contrast, expression of the construct mutated in the phospholipase domain (SWS^S/D^) does not rescue the phenotype. Number of tested flies and s.e.m. are indicated. ***P<0.001. (B,C) Expression of SWS via *loco*-*GAL4* in wild-type flies causes vacuole formation at the first optic chiasm (o.c., arrow), whereas SWS^S/D^ expression via *loco*-*GAL4* does not (C). Both flies were 14 days old. (D,E) Analyzing 14-day-old *loco*-*GAL4;UAS-SWS* flies at the electron microscopic level showed changes in the chiasma glia at the first optic chiasm (D), with multiple membranous whorls and vesicles accumulating in the glial cytoplasm (arrows, E). (F) When inducing SWS^S/D^ with *loco*-*GAL4*, the chiasma glia (g) is indistinguishable from wild type at 14 days. Scale bars in B,C: 12 µm, in D: 2 µm, in E,F: 1 µm. re, retina; la, lamina; me, medulla.

SWS is a 1425 amino acid (aa)-long transmembrane protein with a highly conserved C-terminal domain that mediates its phospholipase function, and a N-terminal region that shows homology to the regulatory subunit of PKA (Kretzschmar et al., 1997). To address the functional importance of these two domains, we previously created constructs with mutations in key residues: SWS^S985D^ carries a mutation in the active site serine in the phospholipase domain and is catalytically inactive (Muhlig-Versen et al., 2005), whereas SWS^R133A^ carries a mutation that prevents its binding to the catalytic subunit PKA-C3 (Bettencourt da Cruz et al., 2008). We also showed that both constructs only partially rescue the neuronal degeneration of *sws^1^* when expressed in neurons. To address the functional requirements of these domains in glia, we expressed them in the glial knockdown. Whereas SWS^S985D^ completely failed to rescue the histological phenotype of 14-day-old *loco*-*GAL4;sws^GD3277^* flies (Fig. 5A), SWS^R133A^ dramatically reduced the vacuole formation (*P*<0.0001). This suggests that the PKA function plays a minor role in glia, whereas they do depend on the phospholipase function. To address whether the degeneration in the SWS^S985D^ rescue experiment might be due to a dominant-negative function of SWS^S985D^, we expressed wild-type SWS and SWS^S985D^ in glia using *loco*-*GAL4* in the wild-type background. Whereas overexpression of wild-type SWS resulted in the formation of vacuoles at the position of the outer chiasma glia in 14-day-old flies (arrow, Fig. 5B), overexpression of SWS^S985D^ had no effect (Fig. 5C). To analyze this in more detail, we prepared electron microscopic sections from 14-day-old flies, which showed that the chiasm glia was dramatically enlarged and contained numerous vacuoles and intracellular membranous whorls when SWS was expressed (Fig. 5D,E). In contrast, these cells looked indistinguishable from wild type in flies overexpressing SWS^S985D^ (Fig. 5F) and we also did not detect phenotypes in other glial cells (in either preparation). Together, these results show that the failure of SWS^S985D^ to rescue the degeneration in the knockdown is due to glial cells requiring the phospholipase function and not a dominant-negative effect of SWS^S985D^. In addition, they reveal that an excess of SWS leads to defects in chiasm glia and that this also depends on phospholipase activity.

### Loss of glial SWS leads to locomotion deficits

**Fig. 6.**
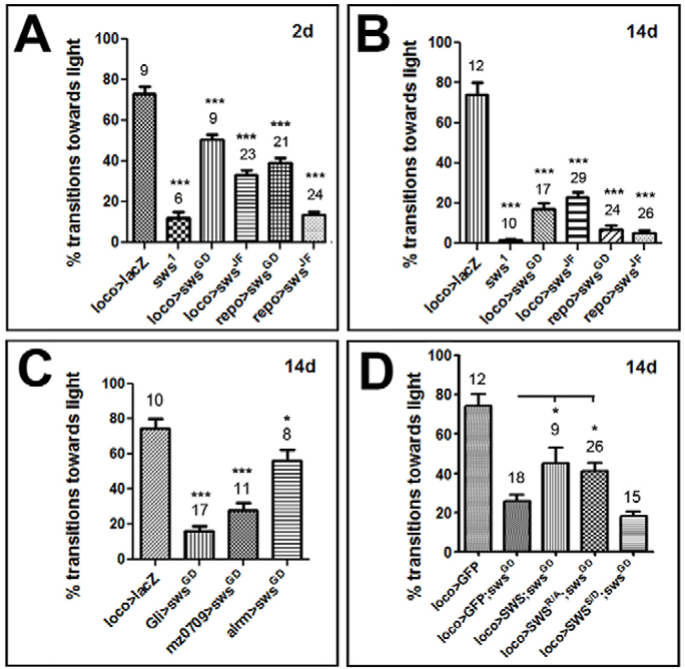
**Loss of SWS in glia causes defects in the fast phototaxis assay.** Pan-glial SWS knockdown flies show less successful transitions in fast phototaxis assays already at 2 days of age (A) that are further reduced when 14 days old (B). (C) Deficits in the fast phototaxis assay are also observed in glial-subtype-specific SWS knockdown flies. (D) Expression of SWS^R/A^ suppresses the phototaxis phenotype in the 14-day-old glial knockdown, whereas SWS^S/D^ does not. Number of tested groups (with five to ten flies) and s.e.m. are indicated. *P<0.05, ***P<0.001.

*sws* mutant flies show locomotion deficits, and mutations or organophosphate-induced changes in NTE cause paralysis and spastic paraplegia in humans. To determine whether the loss of glial SWS and the resulting neuronal wrapping defects contribute to the movement defects, we performed fast phototaxis assays on glial knockdown flies. As shown in Fig. 6A, inducing the *sws^GD3277^* construct with *repo*-*GAL4* or *loco*-*GAL4* resulted in a significant reduction in the performance in this assay as early as in 2-day-old flies, with about 51% and 37% transitions towards light, respectively, compared to 73% in controls (*loco*-*GAL4;UAS-GFP*). Knocking down SWS via the *sws^JF03428^* construct caused an even more severe reduction in performance, with 34% (*loco*-*GAL4*) and 12% (*repo*-*GAL4*) transitions towards light; the latter is not significantly different from *sws^1^* mutants (Fig. 6A). Testing animals at 14 days of age revealed that this phenotype is progressive: all of the knockdown lines, as did the *sws^1^* mutant, performed worse at this age than at the earlier time point (Fig. 6B). We also tested whether the behavioural phenotype occurs when we knock down SWS with the subtype-specific drivers and found that *Gli*-*GAL4;sws^GD3277^* and *mz0709-GAL4;sws^GD3277^* also performed badly when 14 days old (Fig. 6C). Correlating with the weaker histological phenotype, *mz0709-GAL4;sws^GD3277^* flies did better, with 28% transitions towards light compared to 16% for *Gli-GAL4;sws^GD3277^* (*P*<0.05). Using *alrm*-*GAL* for the knockdown, which did not result in a histological phenotype (see Fig. 3A), induced a weak but significant (*P*<0.05) phototaxis phenotype at 14 days of age, with 57% transitions (which is a 23% reduction compared to wild type), suggesting that SWS might play a minor role in astrocyte-like glia.

To confirm that this effect on behaviour is due to the loss of glial SWS and whether the phospholipase function is required, we again performed rescue experiments. As expected, expression of wild-type SWS significantly improved the performance, although it did not restore it to the levels of controls (Fig. 6D). Similar to the results from the histology, although expressing SWS^R133A^ (the mutation in the PKA-binding domain) in 14-day-old *loco*-*GAL4;sws^GD3277^* rescued the behaviour, it was not in fact significantly different from wild-type SWS (Fig. 6D). In contrast, SWS^S985D^ expression did not improve the locomotion deficit, again revealing that the phospholipase function is crucial in glia whereas the PKA function is not. Although we did see a reduction in the fast phototaxis assay in flies expressing SWS^S985D^ via *loco*-*GAL4* in the wild-type background, the same effect was seen when using wild-type SWS or SWS^R133A^ (Fig. S6). That all SWS constructs have this effect strongly suggests that, also in this case, the remaining behavioural deficits in the rescue with SWS^S985D^ are due to this construct not being functional rather than inducing a dominant-negative effect of SWS^S985D^.

**Fig. 7.**
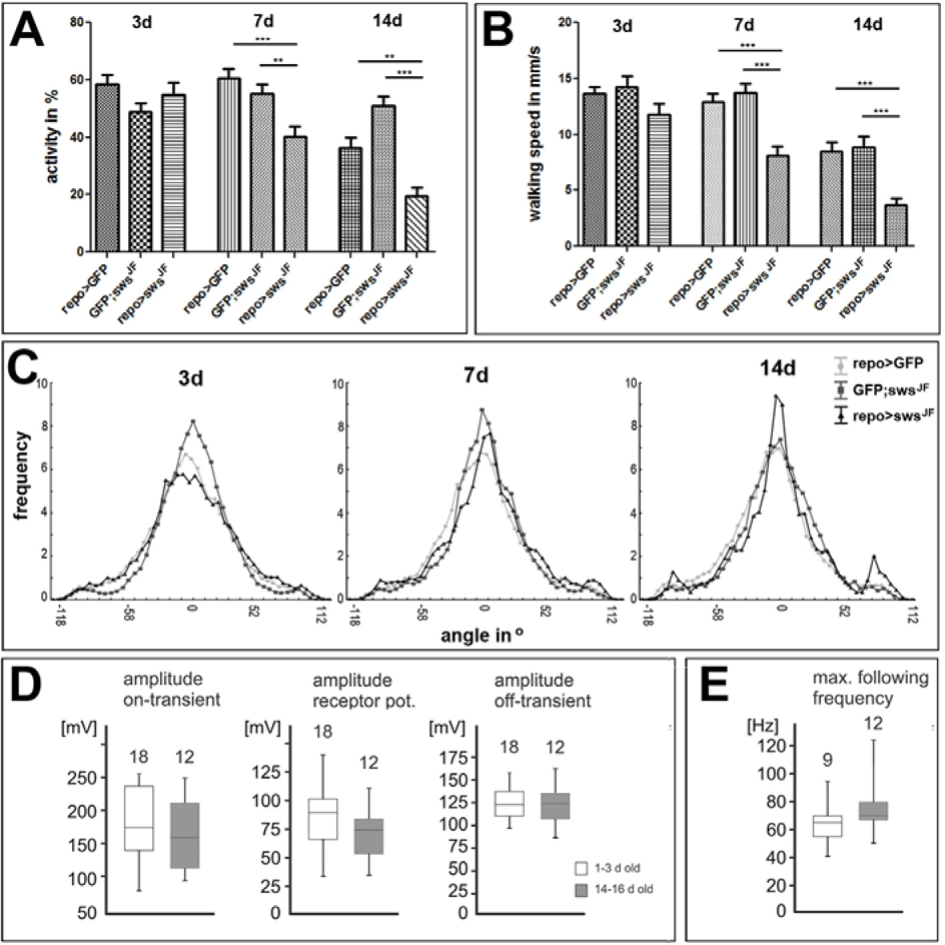
**Loss of glial SWS causes locomotion deficits, whereas the visual input** **is not affected.** Using the Buridan's paradigm, *repo*-*GAL4;sws^JF^* knockdown flies show a decline in activity (A) and walking speed (B) when aged for 7 days or 14 days, compared to controls (*repo*-*GAL4;UAS-mCD8:GFP* and *UAS-mCD8:GFP;sws^JF^*). **P<0.01, ***P<0.001. (C) The knockdown does not affect the orientation of the flies towards the landmark in the Buridan's paradigm. *n*=16 for all genotypes. (D) No statistical differences (Mann–Whitney *U*-test, *P*>0.2) were observed between young and old *repo*-*GAL4;sws^JF^* flies in the amplitude of the on-transient, the compound receptor potential amplitude, or the amplitude of the off-transient. (E) The maximum following frequency in the ERG was similar between young and old SWS knockdown animals. Boxes demark medians and quartiles, and error bars the 10 and 90% values. The number of tested flies is indicated above each bar.

The fast phototaxis test relies on visual input, information processing in the CNS and locomotion. The most prominent phenotype of the glial knockdown is the formation of the membranous structures in the lamina, and this could affect the insulation of photoreceptor neurons and the detection of light in the fast phototaxis assay. We therefore used the Buridan's paradigm to distinguish between visual and processing/locomotion deficits. In this assay, the flies walk back and forth between two inaccessible landmarks in the form of vertical stripes, which they try to keep in a fixed position of their visual field. Analyzing the general activity of *repo*-*GAL4;sws^JF03428^* knockdown flies did not reveal a difference in 3-day-old flies compared to controls but, at 7 days and 14 days of age, the activity of these flies was significantly reduced (Fig. 7A). Measuring the walking speed in 3-day-old knockdown flies showed no significant difference but, when testing at 7 days, they walked significantly slower than controls (Fig. 7B). This was also detectable in 14-day-old knockdown flies when compared to age-matched controls, although aging reduced the walking speed in general (Fig. 7B). In contrast, when analyzing the orientation towards the visual landmark, we did not detect any effects of the knockdown at any age tested (Fig. 7C), showing that the glial knockdown had no major effect on vision.

To further verify that vision was not impaired, we performed electroretinogram recordings (ERGs) on 1- to 3-day-old and 14-day-old *repo*-*GAL4;sws^JF03428^* flies. It has been shown that glial cells, specifically epithelial glial cells, can modulate ERG responses, including the ‘on’ and ‘off’ transients that are due to responses of lamina cells (Heisenberg, 1971; Pantazis et al., 2008). Measuring the responses to a light pulse of 1 s revealed the typical tonic compound potential and neither the amplitude of the on-transient, nor that of the receptor potential, nor that of the off-transient (Fig. 7D) were significantly different in aged knockdown flies. We next tested whether temporal resolution of the visual system was significantly impaired in 14-day-old *repo*-*GAL4;sws^JF03428^* animals by delivering bouts of ten light pulses of 10 ms duration each and increased the pulse frequency in increments of 5 Hz. We then determine the lowest frequency at which not all individual stimuli were resolved as step voltage changes in the receptor potential. Again, the quantification showed that the maximum following frequencies were statistically similar in young and old *repo*-*GAL4;sws^JF03428^* flies (Fig. 7E). In addition, we determined the amplitudes and speeds of adaptation during light stimuli of 20 durations and also found no significant differences (data not shown). That we did not detect any differences in the ERGs further supported the conclusion from the Buridan's paradigm that the knockdown does not impair vision. Together, these experiments suggest that the loss of SWS in glia induces locomotion deficits by interfering with the function of neurons in the CNS that process the visual input and/or execute locomotion.

### The glial knockdown induces defects in neuronal transmission

**Fig. 8.**
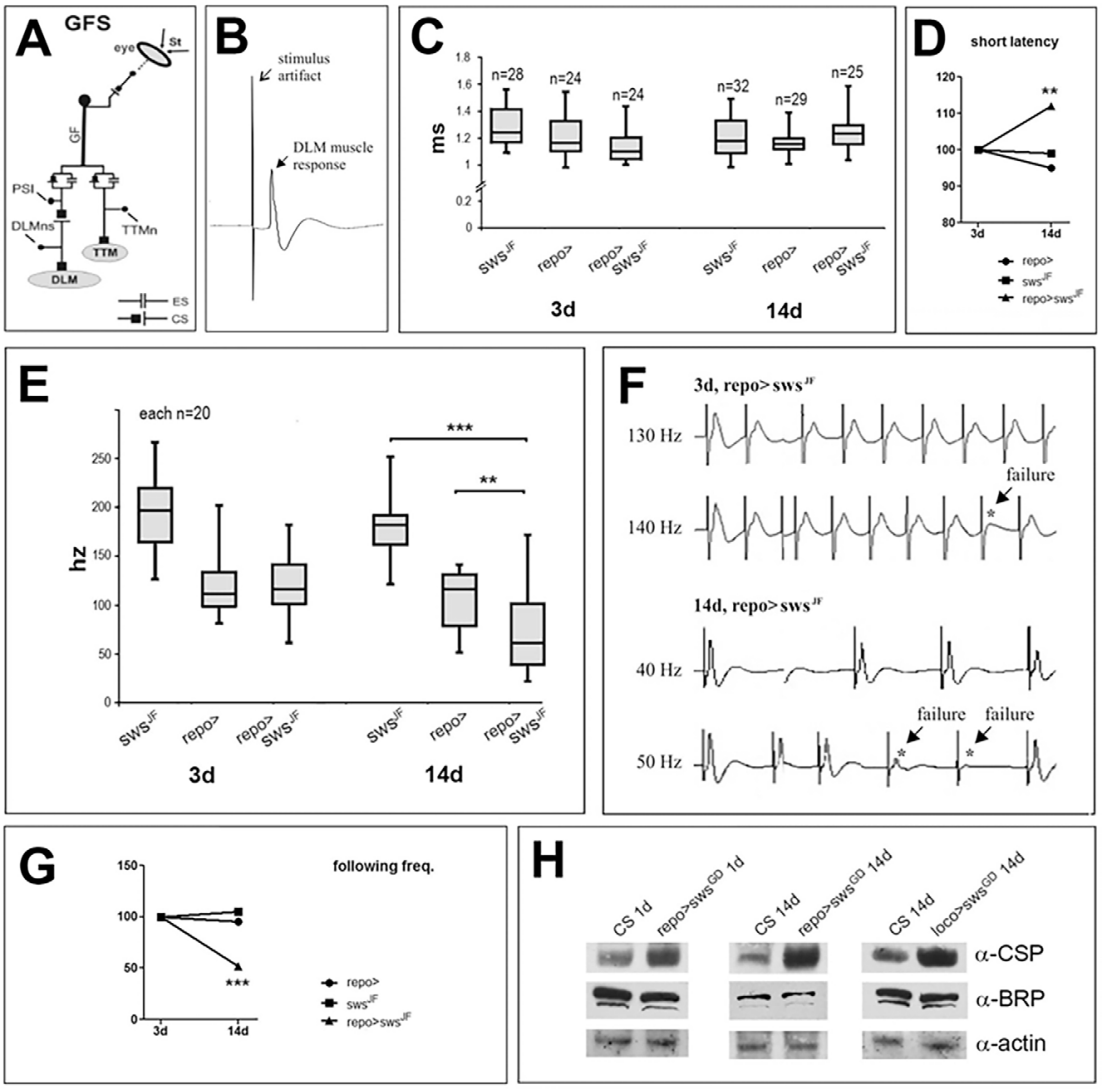
**The glial SWS knockdown reduces the reliability of responses in the GF.** (A) Schematic of the giant fibre (GF) system (GFS). CS, chemical synapse; ES, electrical synapse; DLM, dorsal longitudinal muscle; DLMns, dorsal longitudinal muscle motor neurons; PSI, peripherally synapsing interneuron; St, stimulus; TTM, tergotrochanteral muscle; TTMn, tergotrochanteral muscle motor neuron. (B) Representative recording of a DLM fibre response to electrical GF stimulation. (C) Quantification of response latencies in controls (*repo*-*GAL4* or *sws^JF^* alone) and in glial *sws* knockdowns (*repo*-*GAL4;sws^JF^*) at 3 and 14 days post-adult-eclosion. The glial knockdown of SWS does not affect response latencies when compared to age-matched controls. (D) A small but statistically significant increase in latency was found in 14-day-old *rep*o-*GAL4;sws^JF^* flies compared to 3-day-old ones (Mann–Whitney *U*-test, **P<0.01), whereas controls showed no age-dependent changes (Kruskal–Wallis ANOVA, *P*>0.2). (E) Quantification of maximum following frequency. Whereas young *repo*-*GAL4;sws^JF^* flies follow stimulation frequency as reliably as *repo*-*GAL4* controls, they do show lapses in response already at 50 Hz when 14 days old, whereas the controls do not. **P<0.01, ***P<0.001. Boxes demark medians and quartiles, error bars the 10 and 90% values. (F) Representative traces of muscle responses to bouts of ten GF stimuli at different frequencies at 3 (upper traces) and 14 (lower traces) days post-adult-eclosion. Three-day-old glial *sws* knockdown flies show reliable muscle responses to GF stimulation at 130 Hz and first failures occur at 140 Hz (asterisk). In contrast, the 14-day-old glial *sws* knockdown already shows failures (asterisks) at 50 Hz GF stimulation. (G) Normalized changes in following frequency with aging for *repo*-*GAL4;sws^JF^* flies, *repo*-*GAL4*, and *sws^JF^*. A significant decrease with age occurs only in glial SWS knockdown flies. ***P<0.001. (H) Western blots showing that the levels of cystein string protein (CSP) are increased in *repo*-*GAL4;sws^GD^* flies, whereas Bruchpilot (BRP) levels are unaffected. A loading control using anti-actin is shown below.

To address whether we can detect changes in the activity of a neuronal pathway that relays sensory input to locomotion output, we measured the reliability of action potential conduction and synaptic transmission in the giant fibre (GF) escape response pathway. The GFs are an identifiable pair of interneurons that relay input from the visual system to the thoracic ganglia to trigger a jump response or initiate flight. Whereas the jump response is achieved by a mixed chemical-electrical synapse between the GFs and the tergotrochanteral motor neurons (TTMns), the initiation of flight requires activation of an additional interneuron [the peripherally synapsing interneuron (PSI)] that transmits signals to the dorsal longitudinal muscle (DLM) motor neurons via a cholinergic synapse (Fig. 8A) (Allen and Godenschwege, 2010). First, we tested whether this circuit operated at normal speed by direct electrical stimulation of the GF to measure the delay between GF excitation and flight muscle responses (Fig. 8B). Comparing this short latency response (Kadas et al., 2012) in 3-day-old *repo*-*GAL4;sws^JF03428^* flies with control flies (only carrying the driver or only carrying the RNAi construct) did not reveal significant differences and also aging the mutants for 14 days did not change their response time compared to age-matched controls (Fig. 8C). However, comparing the 3-day-old knockdown flies with 14-day-old ones showed that aging increased the response latency significantly, whereas this was not the case in controls (Fig. 8D). This indicates that the glial knockdown flies show an age-related decline in signal transduction efficiency, correlating with the progressive nature of other phenotypes. However, the net increase of information processing time in this fast-escape circuit was only about 0.1 ms, which might not have significant physiological consequences (this is also probably the reason why the value is still within the range of the controls). Therefore, we next tested whether the reliability to respond to high-frequency stimulation was affected. Applying increasing stimulation frequencies showed that the 14-day-old *repo*-*GAL4;sws^JF03428^* flies were indeed significantly different from both controls (*repo*-*GAL4* or *sws^JF^* alone; Fig. 8E). In addition, this effect was related to aging because older knockdown flies skipped responses at lower frequencies, at which younger ones were not affected (Fig. 8F,G). Although the two control groups performed differently, with *repo*-*GAL4* showing lower following frequencies than *sws^JF03428^* control flies, normalizing the data to the performance of young flies revealed no age effect in the control groups (Fig. 8G). Together, these results suggest that the loss of glial wrapping that we detected in young knockdown flies does not result in detectable changes in the axonal or synaptic transmission, at least in the GF system. However, at 14 days old, the knockdown flies did show a significant reduction of about 50% in the frequency with which reliable information processing occurred.

Because direct stimulation of the motor neurons showed higher following frequencies in all genotypes (Fig. S7), the reduced following frequency through this circuit was not caused by impairments of motor neurons or neuromuscular functions. Direct electrical stimulation of the DLM motor neurons showed shorter latencies of DLM responses than following GF stimulation (Fig. S7B,C), and these increased with age but were not affected by the glial *sws* knockdown (Fig. S7C). Furthermore, DLM following frequencies upon direct motor neuron stimulation were larger than 150 Hz in controls and *sws*-knockdown flies at 3 and 14 days post-eclosion. First DLM failures occurred at 200 Hz of direct motor neuron stimulation in both wild type and the *sws* knockdown (Fig. S7D). No statistically significant differences existed between controls and glial *sws* knockdowns or between young and old flies with regard to the reliability of DLM firing upon motor neuron stimulation (Fig. S7E). By contrast, in 14-day-old glial *sws* knockdown flies, median maximum DLM following frequencies for GF stimulation were about 60 Hz (see Fig. 8F). This suggested that the defect was caused by GF circuit components in the CNS, such as decreased action potential propagation fidelity along the GF axon, or reduced central synaptic function. We therefore determined whether changes in the levels of proteins involved in synaptic vesicle release or recycling could be detected. The cystein string protein (CSP) is part of a chaperone complex that is required for neurotransmitter release, presumably by regulating Ca^2+^-triggered exocytosis (Bronk et al., 2005; Dawson-Scully et al., 2000). Surprisingly, we found that the levels of CSP were increased in both the *repo*-*GAL4-* and *loco*-*GAL4-*mediated knockdown (Fig. 8H). Because the levels of the active zone marker Bruchpilot (BRP), a structural component of the T-bars, was not affected by the glial knockdown (Fig. 8H), the increase in CSP seems to be due to an accumulation of CSP at release sites and not to an increase in the number of release sites. Although future experiments are needed to characterize the effects on synaptic transmission in more detail, these experiments show that the loss of SWS in glia does impair neuronal activity.

## DISCUSSION

Glial cells have been ascribed a variety of support functions but have also recently been suggested to play a more active role in neuronal function (Carmignoto, 2000; Perea and Araque, 2010; Santello et al., 2012). Glial atrophy is detected in several neurodegenerative diseases, and many of the genes connected with these diseases are expressed in neurons and glia. However, their functions in glia and how those functions affect neuronal integrity remains largely unknown (Beirowski, 2013; Lobsiger and Cleveland, 2007). Similarly, NTE activity has been detected in neurons and glial cells in mice (Glynn, 2006, 2007), and SWS expression was detected in some unidentified glial cells in addition to neurons in flies (Muhlig-Versen et al., 2005). However, the function in glia and how this might contribute to the neuronal degeneration and locomotion deficits observed in mutants remained elusive.

Using glia-specific knockdowns, we found that the loss of SWS induces the formation of the large membranous glial structures in the lamina that were previously described in the *sws* mutant (Kretzschmar et al., 1997). This phenotype can be rescued by expression of *Drosophila* SWS or mouse NTE, supporting a conserved function of these proteins in glia. The glial whorls are found in proximity to the basement membrane, where the fenestrated and pseudocartridge glia are localized (Edwards and Meinertzhagen, 2010; Kretzschmar and Pflugfelder, 2002); the latter have been considered to be functionally analogous to subperineurial glia because they express subperineurial-specific markers (DeSalvo et al., 2011). Owing to the position of the glial whorls in the lamina cortex and their formation with drivers expressed in subperineurial glia, the whorls seem to originate from pseudocartridge glia. However, we also detected glial changes in the neuropils, but, whereas the subperineurial/pseudocartridge glia seem to react to the loss of SWS by forming excessive glial processes, the glial cells associated with the neuropil showed stunted glial processes. Because this phenotype occurred in the pan-glial and *mz0709-GAL4*-mediated knockdown and not with *alrm*-*GAL4*, it suggests that the glia responsible for this phenotype is ensheathing glia. That a knockdown in ensheathing glia affects the wrapping of single neurites was at first somewhat surprising because this cell type had been described as ensheathing neuropils but not single neurites (Edwards and Meinertzhagen, 2010; Freeman, 2015). However, inducing mCD4-GFP with this driver revealed that this glial subtype does send processes deep into the optic neuropils and probably does wrap single neurites. Interestingly, we did not detect hyperwrapping, as we observed in the *sws* mutant. Because hyperwrapping appeared neither in the glial knockdown nor in the neuronal knockdown, this phenotype seems to be due to a loss of SWS in both cell types, possibly by interfering with neuronal-glial communication. We previously described that SWS acts as a phospholipase and as a non-canonical PKA regulatory subunit, and that both functions are needed in neurons (Muhlig-Versen et al., 2005). In contrast, glia only seem to depend on the phospholipase function. Because the phospholipase activity regulates phosphatidylcholine, an important component of membranes, loss of SWS could interfere with the extension or maintenance of glial processes by altering the membrane composition of glial cells.

Surprisingly, we found very severe impairments in the fast phototaxis assays already in 2-day-old flies and also in the Buridan's paradigm the flies performed poorly when 7 or 14 days old, confirming that changes in glia contribute to the locomotion defects observed in *sws* mutant flies. However, it should be noted that the glial knockdowns were significantly better than *sws^1^* (*P*<0.05) in the fast phototaxis assays at 14 days, suggesting that the loss of SWS in neurons does contribute to the locomotion deficits. The intact visual orientation and ERGs suggest that the locomotion defects are not caused by a disrupted visual input, and we did not detect changes in motor neuron function. This suggests that the movement deficits are due to changes in CNS neurons that integrate information to orchestrate behavioural output rather than changes in motor neuron output or visual input. Surprisingly, however, defects in neuronal transmission in the GF system could only be detected in 14-day-old knockdown flies and affected the frequency with which the information was reliably processed and not the response time. However, it should be noted that the response time significantly increased with aging in knockdown flies, indicating that age-related changes do occur in the knockdown flies. As mentioned above, our experiments suggest that the behavioural deficits are due to changes in CNS neurons that integrate information. Therefore, the locomotion phenotypes in the fast phototaxis assays observed already at 2 days could be attributed to decreased coding fidelity in the brain of young flies, which is not detected in physiological testing of the GF system. The behavioural defects might also be more dramatic because they rely on the combined effects of several central neuronal circuits, whereas the GF electrophysiology focuses on a small set of neurons. Alternatively, it is possible that the behavioural deficits are caused by an uncoordinated response of neurons, possibly due to the missing modulating or insulating effect of glia on neuronal networks, whereas the transmission within a single neuron is not dramatically affected. Such an uncoordinated activity is supported by the seizure-like behaviour of the glial knockdown flies when startled (see Movies 1, 2). Finally, we investigated whether the reduced reliability in neuronal transmission could be due to deficits in the release of synaptic vesicles or to a reduction in active sites owing to initiation of neuronal degeneration. However, the levels of CSP, which is involved in vesicle release (Burgoyne and Morgan, 2011), were not decreased but actually increased, and the levels of Bruchpilot, an active-site marker (Kittel et al., 2006), were unchanged. Although more experiments are needed to address this issue, the increase in CSP could cause the reduced reliability in the following frequency by increasing vesicle release, thereby eventually resulting in a depletion that prevents a response.

Changes in glia, especially Schwann cells, have been described in many neurodegenerative diseases, including multiple sclerosis, Charcot-Marie-Tooth and hereditary spastic paraplegia (Suter and Scherer, 2003; Timmerman et al., 2013; Waxman, 2006); however, how this impairs neuronal function and integrity is poorly understood. Even in the cases where effects of glial changes on neuronal and axonal degeneration have been investigated, these studies have largely focused on myelination (Beirowski, 2013; Morrison et al., 2013; Nave and Trapp, 2008). However, even then the axonal degeneration might not (or not only) be due to the changes in myelination. Proteolipid protein 1 (*Plp1*)-deficient mutant mice reveal axonal damage and Wallarian degeneration, although fully myelinated (Griffiths et al., 1998). Our results show that the loss of glial SWS does induce neuronal changes and severe locomotion deficits and, owing to *Drosophila* not synthesizing myelin, this provides a valuable system to study the effects of glial changes on neuronal integrity and function independent of myelination. Based on our data in the *Drosophila* model, it will also be interesting to determine whether NTE plays a role in glia in vertebrates, what that role is, and how changes in the glial function contribute to the paraplegia and mental retardation described in humans with mutations in NTE.

## MATERIALS AND METHODS

### *Drosophila* stocks and UAS lines

The *sws^1^* allele was described in Kretzschmar et al. (1997) and the different UAS-SWS lines in Bettencourt da Cruz et al. (2008) and Muhlig-Versen et al. (2005). *repo*-*GAL4*, *UAS*-*lacZ*, *UAS-GFP* and the *sws^JF03428^* RNAi line were obtained from the Bloomington Stock Center. *loco*-*GAL4* was kindly provided by Christian Klämbt (Universität Münster, Münster, Germany), *Gli*-*GAL4* by Vanessa Auld (University of British Columbia, Vancouver, Canada), *Appl*-*GAL4* by Laura Torroja (Universidad Autonoma de Madrid, Spain), and *mz0709-GAL4*, *TIFR-GAL4*, *NP2222-GAL4*, *alrm*-*GAL4* and *mCD4-GFP* by Mary Logan [Oregon Health & Sciences University (OHSU)]. The *sws^GD3277^* RNAi line was obtained from the VDRC Stock Center.

### Tissue sections

Paraffin sections for light microscopy were prepared as described in Bettencourt da Cruz et al. (2005). Briefly, whole flies were fixed in Carnoy's solution, dehydrated and incubated in methyl benzoate before embedding in paraffin. Sections were cut at 7 μm and imaged using the autofluorescence caused by the dispersed eye pigment. Semi-thin and ultrathin Epon plastic sections were prepared as described in Kretzschmar et al. (1997). Semi-thin sections were cut at 1 μm and stained with toluidine blue. Ultra-thin sections were cut at 50 nm and electron microscopic images taken with a FEI Tecnai G^2^ microscope.

### Vacuole measurement

To quantify vacuoles, we photographed the paraffin section that showed the most severe phenotype, without knowing the genotype. Degeneration in the lamina was determined using the threshold discrimination function of ImageJ. For each measurement the total area of the lamina (neuropil and cortex) was selected and the area within that region that fell below a predetermined fluorescent threshold (corresponding to vacuoles) was determined as a percentage of the total area of the lamina. Statistics were performed using GraphPad Prism and one-way ANOVA with a Dunnett's post-test comparing experimental groups to the control.

### Immunohistochemistry

Vibratome sections were prepared as described in Bolkan and Kretzschmar (2014). 50-µm sections were cut on a Leica VT1000 S Vibratome and stained overnight at 4°C with anti-REPO [obtained from the Developmental Studies Hybridoma Bank (DSHB); developed under the auspices of the Eunice Kennedy Shriver National Institute of Child Health and Human Development (NICHD) and maintained by the Department of Biology, University of Iowa] at a dilution of 1:5, or with anti-REPO and anti-activated-caspase-3 (Abgent) at 1:50. Dilutions and blocking steps were done in phosphate-buffered saline with Tween 20 (PBST)+2% goat serum (Jackson ImmunoResearch). Samples were then incubated in secondary antibodies, Cy3- or Cy5-labelled anti-mouse (Vector Labs), at room temperature for 1 h, and used at 1:1000 dilutions to detect REPO. To enhance the caspase signal, samples were incubated with biotin anti-rabbit (1:100) for 2 h at room temperature followed by Streptavidin AF488 (Invitrogen) diluted 1:200 at room temperature for 1 h. Samples were either embedded with polyvinyl alcohol (PVA) antifading mounting medium (Fluka) or Prolong Gold antifade reagent with 4',6-diamidino-2-phenylindole (DAPI). Images were taken with an Apotome 2 (Zeiss) or an Olympus FW1000 confocal microscope.

### Glial cell count

Vibratome sections at the level of the first optic chiasm that showed all optic neuropils were photographed without knowing the genotype. For the optic lobe measurements, we counted all REPO-positive cells surrounding the medulla, lobula and lobula plate neuropils, and, for the epithelial glia, the REPO-positive nuclei within the lamina neuropil were counted before revealing the genotype. Owing to the glial whorl phenotype, the retina was often separated from the lamina in the glial knockdown and we did therefore not count glial cells in the lamina cortex, although this area shows a severe phenotype.

### Western blots

Western blots were performed as described in Carmine-Simmen et al. (2009). Lysates of ten heads were loaded on 7.5% SDS gels and blotted onto PROTRAN nitrocellulose transfer membranes (Whatman). Primary antibodies used were anti-Bruchpilot (BRP, nc82) at 1:250, anti-CSP (ab49) at 1:100, and anti-actin (JLA20, 1:50) as a loading control (all obtained from DSHB). Anti-SWS (used in Fig. S2) was created in our laboratory and is described in Muhlig-Versen et al. (2005). Antibodies were diluted in TBST supplemented with 1% milk powder and incubated overnight at 4°C. Bands were visualized using horseradish-peroxidase-conjugated secondary antibodies (Jackson ImmunoResearch) at 1:1000 at room temperature for 2 h and the SuperSignal West Pico or Femto chemiluminescent substrate (ThermoScientific).

### Fast phototaxis

Fast phototaxis assays were conducted in the dark using the countercurrent apparatus described by Benzer (1967) and a single light source. A detailed description of the experimental conditions can be found in Strauss and Heisenberg (1993). Flies were starved overnight, but had access to water and were tested the following morning. Five consecutive tests were performed in each experiment with a time allowance of 6 s to make a transition towards the light and into the next vial. Flies were tested in groups of five to ten flies. Statistical analysis was done using GraphPad Prism and one-way ANOVA with Dunnett's post-tests.

### Buridan's paradigm

Flies with their wings cut (1 day before testing) were analyzed in an LED arena as described in Götz (1980). Activity reflects the time spent walking during the 15 min observation time in %. The mean walking speed was averaged over the whole observation time in mm/s. The visual orientation capacity of the flies was assessed by comparing all 0.2-s path increments per fly (4500 values in 15 min) to the direct path towards the closer of the two dark stripes presented in the arena. All measured error angles of an individual fly were integrated in a frequency histogram per fly, which was then averaged per group of flies. The Shapiro–Wilk test was used for normal distribution and unpaired *t*-test for significance.

### Electrophysiology

#### Electroretinograms

We used thin-walled microelectrodes (borosilicate) as extracellular electrodes to record compound field potentials from photoreceptors and downstream first order visual interneurons in response to transient light pulses.

#### Giant fibre stimulation

The giant fibre (GF) interneuron can be stimulated in two ways (see Kadas et al., 2012): first, visual neurons upstream of the GF can be stimulated electrically with fine tungsten wires inserted into one eye [long latency response (LLR)]. Second, by increasing the stimulation intensity the GF can be excited directly, thus bypassing neurons upstream to visual interneurons and photoreceptors [short latency response (SLR)]. Circuit output is measured by recording DLM fibre responses extracellularly with tungsten wires. For visual neuron stimulation it takes >3 ms between electrical stimulation and DLM fibre response (LLR). By contrast, upon direct electrical stimulation of the GF, the latency to the muscle response is always <2 ms (Kadas et al., 2012). We always made sure to directly stimulate the GF neuron electrically (SLR) by first evoking a slower LLR and then increasing the stimulation amplitude until the faster SLR was induced. Therefore, we entirely bypassed postsynaptic integration in the dendrites of the GF interneuron. Stimulation of the GF and recording from the muscle monitors the combined result of action potential conduction along the GF axon, mixed electrical/chemical synaptic transmission to peripherally synapsing interneuron (PSI), cholinergic synaptic transmission from PSI onto the dorsal longitudinal muscle motor neuron (DLMn) axon, and glutamatergic synaptic transmission at the neuromuscular junction (NMJ) (see Fig. 8A for GF wiring schematic). To be able to distinguish whether the observed effects of glial *sws* knockdown are caused by defects of central synapses (GF to PSI and PSI to DLMns) or by defects of the neuromuscular synapse (DLMn to DLM fibre), we also conducted experiments with direct stimulation of the DLMn by inserting the fine tungsten stimulation electrodes directly into the mesothoracic neuromere (see Fig. S6A). Direct electrical stimulation of the DLMns and recording from the muscle monitors the combined result of action potential conduction along the DLMns and glutamatergic transmission at the NMJ, which is always <1 ms (Kadas et al., 2012). For both stimulation paradigms (GF and DLMn stimulation), muscle response latency was defined as the time between stimulation and muscle action potential and averaged over five animals in each test group. Maximum following frequency was defined as the maximum stimulation frequency of either the GF or the DLMns for which ten subsequent stimuli produced ten muscle responses. It was determined by increasing the stimulation frequency in increments of 10 Hz until the first failure in a train of ten stimuli was observed in the muscle recording.

Flies were anaesthetized with CO_2_ and glued by their neck to a thin metal wire (20 μm diameter) with UV-light curing adhesive (Glass Adhesive, UV Cure, clear drying, Home Depot) by applying a 10 s UV pulse with a Dental UV curing lamp (Litex 680A Curing Light, Patterson Dental). Flies were allowed to recover for 30 min and then fixed to a micromanipulator by clamping them into a holder, so that the legs and wings were free to move. For GF or DLMn stimulation, a pair of electrolytically sharpened tungsten wires was inserted either into the eyes or the mesothoracic. A third tungsten wire was inserted into the DLM fibre 6, and a fourth wire was placed into the thorax just beneath the scutellum as the reference electrode (Engel and Wu, 1992). Stimuli of 0.15 ms duration were generated with a Grass stimulator. Stimulation amplitude was just above threshold. Threshold was determined by increasing stimulation amplitude from 1 V to up to 10 V amplitude in 1 V increments until the first muscle response was observed. Recorded muscle responses were amplified 100× with a differential AC amplifier (model 1700, A-M Systems) digitized with a Digidata 1200 (Molecular Devices) and acquired and analyzed with Clampex 8.1 software (Molecular Devices).
